# Cancer mutation screening: Comparison of high-resolution melt analysis between two platforms

**DOI:** 10.3332/ecancer.2015.522

**Published:** 2015-04-15

**Authors:** Henry O Ebili, Mohammad Ilyas

**Affiliations:** 1 Morbid Anatomy and Histopathology Department, Olabisi Onabanjo University, Ago-Iwoye, Nigeria; 2 University College Hospital, Ibadan, Nigeria; 3 Division of Pathology, University of Nottingham, Queen’s Medical Centre Campus, Nottingham, UK

**Keywords:** cancer mutation screening performance, HRMA platforms, ABI 7500, LightScanner

## Abstract

High-resolution melt analysis (HRMA) is a cheap and reliable post-polymerase chain reaction (PCR) cancer mutation screening technique, which is fast gaining clinical relevance. The HRMA capabilities of the LightScanner (Idaho Technology) have been severally studied. However, the ABI 7500 HRM has not been tested against the purpose-built HRM instrument such as the LightScanner.

DNA from formalin-fixed, paraffin-embedded gastric cancer, colorectal cancer, and normal tissue as well as from colorectal cancer cell lines were amplified at exons 2, 3, and 4 of KRAS, and at exons 11 and 15 of BRAF in the ABI 7500 fast real-time PCR machine and subjected to melting both on the ABI and on the LightScanner. HRMA data were analysed with the ABI HRM software v2.0.1 and the LightScanner Call-IT 2.5. We tested the ABI 7500 HRM for internal precision, accuracy, sensitivity, and specificity at mutation screening relative to the LightScanner, using crude percentage concordance, kappa statistics, and the area under the receiver operator characteristics (AUROC) curve on SPSS version 19.

The results show that the ABI 7500 HRMA has a high internal precision, and excellent concordance, sensitivity, and specificity at mutation screening compared with the LightScanner. However, in contrast to the LightScanner HRM software analysis, the ABI HRM software v.2.0.1, cannot distinguish real from certain pseudovariations in PCR amplicons that are sometimes brought about by the artefacts of the melting process.

In conclusion, the ABI HRM has a comparable performance level with the LightScanner, although in certain respects mentioned previously, the LightScanner has an edge over the ABI.

## Background

High-resolution melt analysis is a highly reliable PCR-based technique which has found use in genotyping and mutation scanning and is fast gaining clinical relevance [1, 2].

There are two classes of instruments utilized for HRMA, namely real-time PCR instruments with HRM modules and HRM instruments without PCR capabilities [1, 3]. In the HRM instruments such as the LightScanner, PCR is carried out in a thermocycler/qPCR instruments, and amplicon melting is done in the HRM machines [4]. The captured melting data are thereafter analysed with instrument-specific software. In the real-time instruments such as the ABI 7500 fast real-time PCR machine, PCR and amplicon melting are carried out as a closed tube procedure. The data obtained are analysed with HRM software specific for the instruments [5].

A few studies have compared the performance of HRM across different platforms. Generally, the HRM instruments have a higher discriminating power than the real-time PCR instruments which incorporate HRM modules [1, 3, 6]. In particular, the LightScanner has been shown to have similar sensitivity and specificity as different sequencing methods [7–10]. However, the HRM capabilities of the ABI 7500 real-time PCR has little been tested against the HRM instruments.

The aims of this study are to determine the degree of concordance between the ABI 7500 real-time PCR and the LightScanner in mutation screening and to compare the sensitivity and specificity of the ABI 7500 fast real-time PCR HRMA relative to that of the LightScanner. Our hypotheses are that there is a high rate of concordance between the HRMA results of the ABI 7500 real-time PCR and the LightScanner, and the ABI fast real-time instrument has an excellent sensitivity and specificity relative to the LightScanner. To test these hypotheses, the HRM results obtained with these instruments were analysed with the appropriate statistical tests of agreement. Furthermore, the sensitivity and specificity of the ABI instrument were compared using the area under the receiver operator characteristics (AUROC) curve and manually calculated from the results of HRMA. The results are presented in this article.

## Materials and Methods

This study was carried out at the Division of Pathology, University of Nottingham, Queen’s Medical Centre Campus, Nottingham, United Kingdom.

We tested intra-assay precision or reproducibility of each of the HRM modules, and then, the inter-assay agreements among the modules.

### DNA samples

DNA from clinical gastric cancer, colorectal cancer and normal (all formalin-fixed, paraffin-embedded) tissues, and colorectal cancer cell lines (RKO, DLD1, and HCT116) were included in this study.

### Polymerase chain reaction and amplicon melting

Different genomic regions were amplified using the recently described two-stage PCR protocol (manuscript submitted) in the ABI 7500 fast real-time instrument. PCR was carried out in a 10 μL volume made up of 5 μL of Diamond Hotshot master mix, 1 μL of LC Green Plus dye, 0.5% of Tween, 300 nm of primers, and 2 μL of amplicon-enriched DNA template.

The samples were amplified in singles (for inter-assay testing) and duplicates (for intra-assay testing), and PCR was followed by melting in the ABI 7500 and then in the LightScanner. The primer sequences, cycling and melting parameters used in this study are shown in Tables 1 and 2.

The melting data were analysed with the HRM software v.2.0.1 (ABI 7500) and the LightScanner software Call-IT 2.5. The LightScanner data were analysed with the Amplicon Scanning (LSAS) and the Amplicon Genotyping (LSAG) programmes of the software. In some instances, the sensitivity levels of the LSAS were adjusted until the curves of all the wild-type samples (normal DNA), and other samples tightly clustering with them were identified as a single variant.

### Statistical analyses

Intra-assay and inter-assay agreements were tested for each HRM operation using the crude percentage concordance and kappa statistics in the SPSS version 19. The sensitivity and specificity of the ABI HRMA in detecting mutations relative to the LightScanner were derived from the coordinates of the area under the receiver operating characteristic (ROC) curve and confirmed by manual calculations from the cross tabulations of the HRM results.

### Strategy

DNA samples from cancer and normal tissue were amplified in five gene regions: exons 15 and 11 of BRAF, and exons 2, 3, and 4 of KRAS. For the purpose of comparison of assays, each amplified gene region was treated as a sample (a gene region sample), melted with ABI and then, the LightScanner, and analysed with the ABI HRM software, LSAS and LSAG. For example, when a single DNA sample is experimentally amplified at BRAF 15, BRAF 11, KRAS 2, and KRAS 3, it is regarded as four (gene region) samples and statistically analysed as such. Different experiments were performed for the intra-assay testing than for the inter-assay tests. For the intra-assay agreement, DNA samples were amplified in duplicates at KRAS 2 and KRAS 4 up to a total of 53 gene region samples and melted on the ABI and LightScanner. The degrees of agreements between the duplicates were compared for ABI, LSAS, and LSAG using the statistical tests mentioned earlier. For the inter-assay agreement, DNA samples were experimentally amplified in singles at BRAF 15, BRAF 11, KRAS 2, and KRAS 3 up to a total of 292 gene region samples. The degrees of agreement among the ABI, LSA, and LSAG were also compared using the same statistical tests mentioned.

## Results

### Test of reproducibility of the ABI 7500 real-time fast PCR and the LightScanner

Exons 2 and 4 of KRAS were amplified in some of the above-mentioned samples to a total of 53 duplicate gene regions and subsequently melted on both instruments. Up to eight different amplicon variants were identified by each assay per gene region amplified. For each gene region sample, the duplicate HRMA data were input into SPSS as two different variables (first and second variant calls) for each assay. Statistical analyses with crude percentage concordance and kappa statistics were performed to determine the precision (reproducibility) of each of the assays. The results showed that while each of the assays had excellent intra-assay agreement (ABI: 90.6%, *К* = 0.811 [*P* < 0.001]; LSAS: 96.2%, *К* = 0.920 [*P* < 0.001]; LSAG: 94.3%, *К* = 0.884 [*P* < 0.001]), the LSAS and LSAG had better intra-assay precision or reproducibility.

### Inter-assay agreement between the ABI and the LightScanner

**Figure 1.**
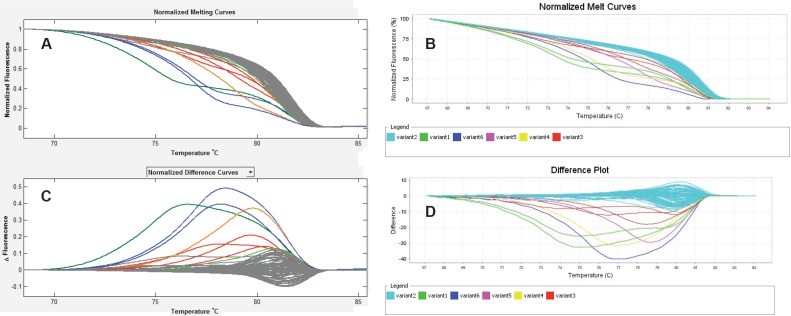
Inter-assay agreement. HRMA of exon 15 of BRAF using the LightScanner (A & C) and the ABI 7500 fast real-time (B & D) platforms showing highly reproducible results between both platforms seen here as identical Normalised Melt Curves (A & B) and mirror-image difference plots (C & D). The non-grey curves in A and C, and the non-blue curves in B and D are the same aberrantly melting samples, while the grey curves in the LightScanner and blue curves in the ABI output represent the same normally melting samples. The LightScanner normalized difference curves output, by default, is inverted relative to the ABI difference plots output.

About 292 gene region samples were amplified in singles at exons 11 and 15 of BRAF, and exon 3 of KRAS. The HRMA data of each sample were entered into SPSS as either wild type or mutant for each assay, irrespective of the number of variants identified by each assay. Then, crude percentage concordance and kappa statistics were utilised to test for agreement among ABI HRM, LSAG, and LSAG. The results showed that the agreements among the three assays were outstanding (ABI versus LSAS: 97.6%, *К* = 0.864 [*P* < 0.001]; LSAG versus ABI: 97.6%, *К* = 0.850 [*P* < 0.001]; LSAS versus LSAG: 97.9%, *К* = 0.878 [*P* < 0.001]) (Figure 1).

### Performance of the ABI and the LSAG relative to LSAS

The area under the receiver operating characteristics (AUROC) was derived for ABI (relative to LSAS and LSAG) and LSAG (relative to LSAS). Table 3 shows the areas and coordinates of the curve for the different HRM assays. Both the ABI HRMA and the LSAG show excellent sensitivity and specificity relative to each other and to the LSAS.

The above results were confirmed by manual calculations of sensitivity and specificity from the cross-tabulations of the results from the three HRMA assays (Tables 4 and 5).

## Discussion

Although the intra-assay agreement for all three assays were outstanding, the crude percentage concordance, and kappa values show that the LightScanner assays have more internal consistency than the ABI 7500 HRM assay. This better internal consistency of the LightScanner is in agreement with a previous study which showed that HRM instruments have a better precision than non-HRM instruments [1, 3].

In this study, we have used the LightScanner as the standard to compare the inter-assay reproducibility and performance of the ABI because the LightScanner has been severally shown to have similar performance levels for mutation detection as the Sequencing methods [7–10]. The three assays show excellent inter-assay agreement, although both LightScanner HRM modules have better inter-assay agreement between them than either has with the ABI. This outstanding inter-assay reproducibility across HRM platforms have similarly been shown among HRM instruments and qPCR instruments with HRM capacity [9, 11]. This simply attests to the robustness of HRMA as a mutation screening method.

The sensitivity of 83.3% of the ABI HRM module at mutation detection found in this study, although excellent, is lower than the 92–100% sensitivity obtained by ABI HRM relative to sequencing in the detection of micro-organisms [12]. However, the specificity of 98.1% is comparable to what was obtained (98.36–100%) in the aforementioned study.

**Figure 2.**
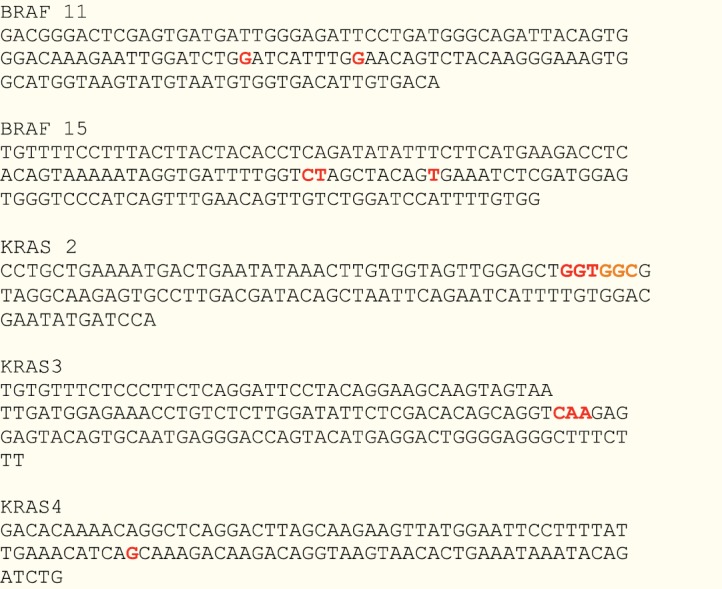
Sequences amplified in this study with mutation hotspots. All the bases in orange and red colours represent mutation hotspots in the gene regions amplified. The red bases shown in KRAS exon 2 represent codon 12 of KRAS, while the orange bases represent codon 13. The two colours used here are intended to differentiate codons 12 and 13 of KRAS.

The 80% sensitivity obtained with the LSAG relative to the LSAS analysis in mutation scanning was expectedly low, considering that LSAG is designed for genotyping, not scanning [4]. We have utilized LSAG module in the analysis of the melting data of amplicons that are 105–145 bp long and that have skewed mutation spots (Figure 2). This is different from the recommendation of the LightScanner manufacturer that LSAG be used for analysing melting data of amplicons designed to be 50–100 bp long with the mutation spot at the middle of the amplicon length. However, the degree of inter-assay agreement between the LSAS and LSAG analyses and sensitivity level of 80% obtained in this study shows that LSAG may just as well be suitable for mutation scanning as LSAS. This is more so when the specificity of the LSAG at mutation detection as found in this study is 100%. Furthermore, the high degree of overlap of the sensitivity and specificity of the LSAG and the ABI 7500 as determined by the AUROC curve lends support to the suggestion that LSAG may also be used for mutation scanning.

**Accession numbers of DNA sequences shown in** Figure 2

- BRAF exon 11 [NCBI Reference Sequence NC_018918.2]
- BRAF exon 15 [NCBI Reference Sequence NC_018918.2]
- KRAS exon 2 [NCBI Reference Sequence NC_018923.2]
- KRAS exon 3 [NCBI Reference Sequence NC_018923.2]
- KRAS exon 4 [NCBI Reference Sequence NC_018923.2]

Our study did not include any sequencing methods as the aim is to show the extent to which the ABI HRMA replicates the LightScanner HRMA at mutation screening. This is more so as HRMA has severally been shown to have similar sensitivity and specificity as sequencing at mutation detection [7–10]. We have applied the stringent measures for adequate PCR amplification, that is, cycle threshold value of 30 or less and a normal amplification curve in order to ensure robust HRMA results [13].

**Figure 3.**
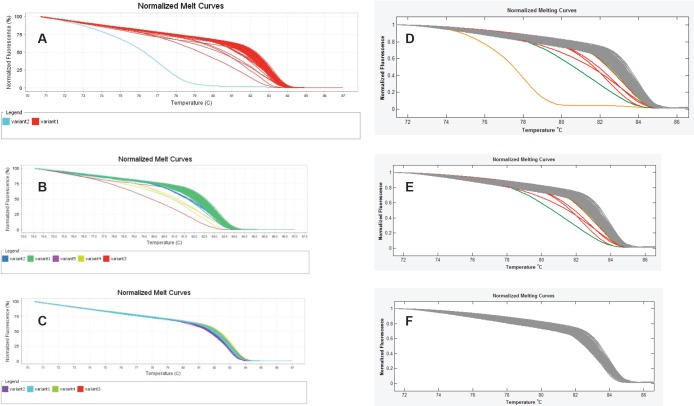
‘Unstable’ variant calls by the ABI HRM software v2.0.1. A, B & C. Sequential exclusion of aberrant variants from analysis with the ABI results in variant call changes of the remaining samples. D, E & F. Sequential exclusion of samples with aberrant melting patterns from analysis with the LightScanner Call-IT 2.5, however, did not change the original calls of the remaining samples.

**Figure 4.**
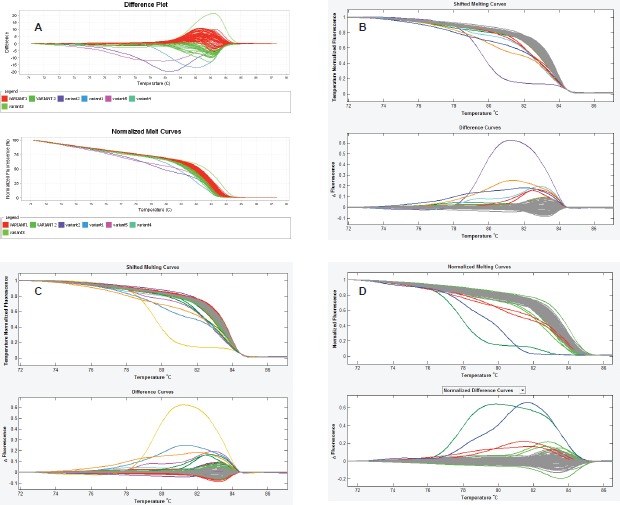
Spurious variant calls by the ABI HRM software. A. In this set of experiment, the ABI HRM software identified two large clusters of samples as belonging to two different melting groups (red and green curves) even though both clusters merge snugly and contain wild-type controls. Furthermore, the following assignment of normal samples from both clusters as wild-type controls, the software still recognised them as different. B & C. The amplicon scanning programme of the LightScanner Call-IT 2.5 (LSAS) also recognized some samples from same cluster as different. However, when the sensitivity level of the programme was adjusted, the software correctly classified the samples as one variant (grey). D. The amplicon genotyping programme (LSAG) correctly classified the samples as the same variant (grey), even without adjustment ofthe sensitivity.

Finally, we observed that the LightScanner software analysis is superior to that of the ABI 7500 HRMA in two respects. The first is that the variant calls by the LightScanner software are ‘stable’. That is, they are not affected by the addition or subtraction of a sample with a deviant melting pattern to a set of samples. In contrast, analysis by the ABI HRM software is more ‘delicate’ and its variant calls are significantly affected by the addition or subtraction of one or more samples with different melting patterns (Figure 3). The second respect in which the LightScanner software analysis is more robust than that of the ABI 7500 is in the capacity of the LightScanner analysis to accommodate certain subtle artificial variations in amplicons that are brought about by the vagaries of the melting process. For example, instruments used for HRMA are known to show some variations in melting temperature, *T*_m_, during amplicon melting. These variations are worse with the qPCR instruments incorporating HRM (including some of those manufactured by ABI) than the purpose-built HRM instruments. Variations in *T*_m_ can result in false variant calls by the instruments [2]. While the LSAG module can accommodate such artificial variants by default, the LSAS module can be adjusted to accommodate them using the sensitivity level of the software. This capability is lacking with the ABI HRM v2.0.1 software (Figure 4).

## Conclusion

The ABI 7500 HRMA shows a high internal precision, and excellent concordance, sensitivity, and specificity at mutation screening compared with the LightScanner. In addition, the LightScanner HRM software analysis, but not the ABI HRM software v.2.0.1, can nullify certain pseudovariations in PCR amplicons brought about by the artefacts of the melting processes.

## List of Abbreviations

HRMA: High-resolution melt analysis
LSAS: LightScanner amplicon scanning programme
LSAG: LightScanner amplicon genotyping programme

## Declaration of Interest

The authors declare no conflict of interest.

## Author’s Contributions

HOE: Experiment development, execution and analysis, manuscript drafting, editing and preparation of the final manuscript version. MI: Conception, experimental analysis, manuscript editing, and preparation of final version for publication.

## Figures and Tables

**Table 1.** Sequences of primer pairs used in the study.

Gene region	Sequences
BRAF exon 11	F: 5’-GACGGGACTCGAGTGATGAT-3’ R:5’-TGTCACAATGTCACCACATTACA-3’
BRAF exon 15	F: 5’-TGTTTTCCTTTACTTACTTACTACACCTCA-3’ R: 5’-CCACAAAATGGATCCAGACA-3’
KRAS exon 2	F: 5’-CCTGCTGAAAATGACTGAATATAA-3 R: 5’-TGGATCATATTCGTCCACAAAA-3’
KRAS exon 3	F: 5’-TGTGTTTCTCCCTTCTCAGGA-3’ R: 5’-AAGAAAGCCCTCCCCAGT-3’
KRAS exon 4	F: 5’-GACACAAAACAGGCTCAGGACT-3’ R: 5’-CAGATCTGTATTTATTTCAGTGTTA-3’

**Table 2.** Cycling parameters for utilized for amplification of genes in this study.

Parameters		Temperature	Time
Initial denaturation		95°C	3.21 min
40–45 cycles	Denaturation	95°C	10 sec
	Annealing	55°C	45 sec
	Extension	72°C	30 sec
Final extension		72°C	5.20 min
Melt curve (ABI 7500) Melt curve (LightScanner)		95°C	15 sec
		60°C	1 min
		95°C	15 sec
		60°C	1 min
		68.3°C–95.4°C Auto exposure	

**Table 3.** AUROC analysis for HRMA results of ABI and LightScanner.

Assay	Area	Standard error	95% confidence interval	*P*	Sensitivity	1 specificity
ABI (LSAS)*	0.913	0.04	0.834–0.992	<0.001	0.833	0.008
ABI (LSAG)*	0.949	0.033	0.884–1.000	<0.001	0.917	0.019
LSAG (LSAS)*	0.90	0.044	0.814–0.986	<0.001	0.800	0.000

* Analysis of performance of ABI was done relative to LSAS and LSAG, and LSAG relative to LSAS.

**Table 4.** Cross tabulation of results of mutation screening by ABI and LightScanner.

		**LSAS**		
ABI		**Wild type**	**Mutant**	**Total**
	Wild type	260	5	265
	Mutant	2	25	27
	Total	262	30	292
		**LSAG**		
ABI		**Wild type**	**Mutant**	**Total**
	Wild type	263	2	265
	Mutant	5	22	27
	Total	268	24	292
		**LSAS**		
LSAG		**Wild type**	**Mutant**	**Total**
	Wild type	262	6	268
	Mutant	0	24	24
	Total	262	30	292

**Table 5.** Manual calculation of performance measures of the ABI and LightScanner.

Assay	Sensitivity	Specificity	Accuracy
ABI (LSAS)	83.3%	98.1%	97.6%
ABI (LSAG)	91.7%	99.2%	97.9%
LSAG (LSAS)	80%	100%	97.6%
